# Measuring Activity Levels at an Acute Stroke Ward: Comparing Observations to a Device

**DOI:** 10.1155/2013/460482

**Published:** 2013-10-27

**Authors:** Sharon F. Kramer, Toby Cumming, Leonid Churilov, Julie Bernhardt

**Affiliations:** ^1^Stroke Division, Florey Neuroscience Institute, 245 Burgundy Street, Heidelberg, Melbourne, VIC 3084, Australia; ^2^School of Physiotherapy, La Trobe University, Melbourne, VIC 3086, Australia

## Abstract

*Background*. If a simple system of instrumented monitoring was possible early after stroke, therapists may be able to more readily gather information about activity and monitor progress over time. Our aim was to establish whether a device containing a dual-axis accelerometer provides similar information to behavioural mapping on physical activity patterns early after stroke. *Methods*. Twenty participants with recent stroke ≤2 weeks and aged >18 were recruited and monitored at an acute stroke ward. The monitoring device (attached to the unaffected leg) and behavioural mapping (observation) were simultaneously applied from 8 a.m. to 5 p.m. Both methods recorded the time participants spent lying, sitting, and upright. *Results*. The median percentage and interquartile range (IQR) of time spent lying, sitting, and upright recorded by the device were 36% (15–68), 51% (28–72), and 2% (1–5), respectively. Agreement between the methods was substantial: Intraclass Correlation Coefficient (95% CI): lying 0.74 (0.46–0.89), sitting 0.68 (0.36–0.86), and upright 0.72 (0.43–0.88). *Conclusion*. Patients are inactive in an acute stroke setting. In acute stroke, estimates of time spent lying, sitting, and upright measured by a device are valid.

## 1. Introduction 

The health benefits of physical activity are well established. It is known that physical activity reduces the risk of stroke [1, 2]. Additionally, lower levels of physical activity are related to a higher risk of cardiovascular mortality [3, 4]. After stroke physical activity takes on even greater importance for improving function and independence in activities of daily living. Higher levels of physical activity in stroke survivors have shown to be related to better quality of life [5]. Intensive physiotherapy input may enhance the rate of recovery and can have a favourable effect on a person's ability to perform activities of daily living within the first six months of stroke [6–9]. An early start of mobilisation in acute stroke units can improve outcomes [10, 11]. However, not much is known about how early and how active stroke survivors need to be to optimize recovery. 

There are several methods available to monitor physical activity. One method that is used extensively in stroke samples is behavioural mapping. A recent review identified 15 studies that used behavioural mapping to record activity throughout the day in hospitalized stroke patients [12]. Behavioural mapping is a structured observational method that requires a researcher to intermittently observe a participant at set intervals over a period of time. While it is a rich source of data and has been shown to be reliable [13, 14], the method is very time consuming and costly. Monitoring participants' activity levels using instrumented methods is becoming more commonly applied in stroke studies [15]. Using an accelerometer based device to record activity patterns is far less time consuming than behavioural mapping and it has been proposed as a practical alternative for monitoring physical activity [15, 16].

Across the different studies that measure physical activity levels in stroke survivors, few have focussed on the acute stage [14, 17–21]. If a simple system of instrumented monitoring was possible early after stroke, therapists and researchers may be able to more readily gather baseline information about activity and monitor progress with physical rehabilitation over time. The information could also be used to give feedback to stroke survivors, creating greater interest and involvement in their rehabilitation. 

The definition of physical activity often varies according to the method used to measure it. Furthermore, it is often described in terms of intensity or step counts. Since it is known that in an acute hospital stroke survivors spent the majority of time lying down [14, 21], measures of intensity and step counts are less relevant. We argue that in acute stroke, it is important to distinguish between lying, sitting, and an upright position (i.e., standing or walking). The ability to sit requires substantial postural control, which can be very challenging after stroke and therefore represents a higher level of functional ability than lying down. The fact that sitting represents a functional ability is reflected in the scoring hierarchy of several functional outcome measures such as the Mobility Scale for Acute Stroke [22] and the Barthel Index [23, 24]. In the hierarchy of these measures, patients get a better score when they are able to sit. This is relevant since these measures are predictive of outcome after stroke [25–27].

We aimed to (1) describe activity levels in an acute stroke setting, (2) determine agreement between a well established observational method (behavioural mapping) and an accelerometer based device regarding physical activity and body position, and (3) establish the acceptability of the device to the participants.

We hypothesised that

1. the device would give similar information compared to behavioural mapping about the percentage of time spent lying, sitting, and upright (standing and walking), in acute stroke survivors,
2. the device would be readily accepted by participants early after stroke.

## 2. Materials and Methods

### 2.1. Study Design

This is a comparative study of two methods of measuring physical activity. Measurements occurred over a single day from 8 a.m. to 5 p.m., the most active part of a participant's day, within two weeks of stroke. 

### 2.2. Participants

Participants were recruited from an acute stroke care ward of a large metropolitan hospital in Melbourne, Australia. All stroke survivors >18 years of age with confirmed stroke within the previous 14 days were eligible, with the exception of those receiving palliative care or those with severe pre-morbid disability (modified Rankin Score = 5) [28]. The primary objective of this study was to compare activity across the two methods in a representative sample of participants who spanned the stroke severity spectrum. We aimed to include a sample of 20 participants that represented a broad range of stroke severity and balanced in gender. Recruitment of a convenience sample of 25 participants allowed for an attrition rate of 20%. 

All participants or their nominated representative received written and oral information about the research and provided informed consent prior to participation in this study.

### 2.3. Measures of Physical Activity

*Observation.* We used a method of behavioural mapping that was developed and tested in an acute stroke population and has been shown to be reliable and acceptable to stroke survivors [14, 21, 29]. Using this method, participants are observed at 10-minute intervals, except for four randomly scheduled 10-minute breaks, over a 9 hour day. When participants were out of view (e.g., in the bathroom or off the ward for tests) they were marked as unobserved. A trained observer recorded activity using a standardized form that included 15 prespecified activities. These activities were grouped into the categories of interest: lying, sitting, standing, and walking. In this study we defined three levels of physical activity: lying, sitting, and upright (a combination of the recorded data of standing and walking activity). 

**Figure 1:**
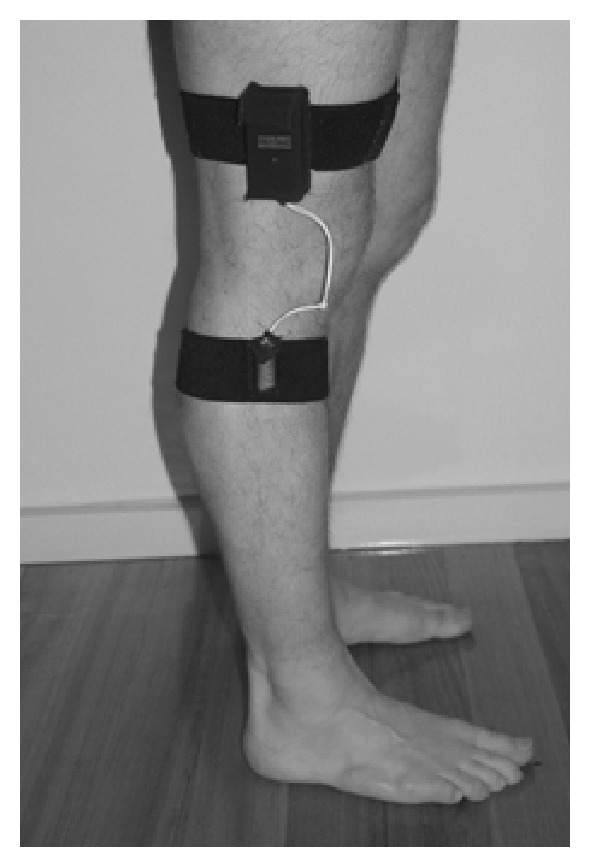
Attachment of the PAL2 on the upper and lower leg.

*Device.* The device was the PAL2 (Gorman ProMed Pty. Ltd). It is based on a dual-axis accelerometer, combined with tilt switches, and samples at a rate of 10 Hz. It has been shown to be a valid device to record physical activity in Chronic Obstructive Pulmonary Disease [30]. Earlier versions of the PAL2 have been shown to be valid in people with a hip fracture [31] and an elderly population [32]. It registers composite information of tilt and movement. The PAL2 is worn on the lateral side of one leg, attached with two straps above and below the knee. The combination of information from both of the tilt switches, one attached to the upper leg and one on the lower leg (Figure 1), allows the wearer's position (lying, sitting, or upright) to be determined. The device provides actual time spent (seconds) in each activity and the time at which the activity occurred.

The PAL2 software was downloaded onto a laptop. The software is needed to initialise the device to start data recording, to download data from the device after recording, and to calibrate the data. After initialising, the device was attached to the unaffected leg.

### 2.4. Acceptability of Wearing the PAL2

At the end of the recording day all participants were asked to rate their experience of wearing the device. Participants scored their agreement with the statement “Wearing the device on my leg was comfortable” on a 5-point Likert-scale ranging from “strongly agree” to “strongly disagree.” In addition, any comments of participants or staff about the device were recorded.

### 2.5. Other Prespecified Information

Demographic information that allowed the sample to be described was gathered. This included age, gender, pre-morbid living conditions, premorbid disability level (modified Rankin Scale score) [28], stroke side, stroke type (Oxfordshire classification) [33], and severity (NIHSS) [34].

### 2.6. Data Processing

For this study the recorded data were classified into three categories: time spent lying, time spent sitting, and time spent upright.

*Observation.* For each participant up to 54 observations were taken during behavioural mapping and details were recorded on standardized forms. The forms were scanned and data were automatically transferred into an Access database (Microsoft Access 2010). The data could then be transformed into the categories of interest (lying, sitting, and upright). The percentage of time spent in each category was calculated by dividing the counts per category by the total number of observations for each participant. For example a participant who was observed sitting for 22 of the 49 completed observations was judged as sitting for 45% of the day (22/49 × 100). The total number of observed time samples did not include the four planned observer breaks or time samples when the participant could not be observed. 

*Device.* Once data from the PAL2 were downloaded via a USB-port on to a computer using the PAL2 software, a raw data graph was generated. In this graph the activity of the participant is represented by a trace for position and a trace for movement. The position of the trace on the graph (high or low) is derived from the combined information of the tilt switches and can be used to determine the different positions of the participant (lying, sitting, or upright). Calibration of the data involved setting thresholds to distinguish sitting from standing and sitting from lying using the values on the graph. To distinguish between standing still and moving (i.e., walking) we used the default intensity setting of the device. After calibration the time in each category in minutes was automatically calculated and for this study we calculated the time spent upright by summing time spent standing and moving. Percentages of time spent in each category were calculated by dividing the time spent per category by the total recording time (maximum of 540 minutes) for each participant. For example, 230 minutes of sitting out of a total time of 540 minutes was judged as sitting for 43% of the day (230/540 × 100).

### 2.7. Statistical Analysis

STATA (version 11) was used to analyse the data. Percentages of the day spent lying, sitting, and upright were estimated for each participant for both behavioural mapping and PAL2. To investigate the agreement between the two measurement methods, we conducted a paired analysis to calculate correlation between the two measurements using Intraclass Correlation Coefficients (ICC) [35] and Lin's Concordance Correlation Coefficients (CCC) [36]. To indicate the strength of agreement we used the following classification : <0.00 = poor, 0.00–0.20 = slight, 0.21–0.40 = fair, 0.41–0.60 = moderate, 0.61–0.80 = substantial, and 0.81–1.00 = almost perfect agreement [37].

 Additionally we calculated a reduced major axis regression. This is particularly suitable for scenarios when both measuring devices or methods produce readings not free from measurement error [38]. Unlike other methods for agreement assessment (such as Bland-Altman limits of agreement [39]), reduced major axis regression allows disagreement to be separated into fixed and proportional components. The two methods can provide readings that differ by a consistent amount across magnitude (fixed bias) or that differ by a changing amount across magnitude (proportional bias). One example of proportional bias is for agreement to decrease as readings increase in magnitude; in this case the slope of the reduced major axis regression line will differ from that of the line of “perfect agreement” (slope = 1) [40, 41]. 

To further investigate whether the patterns of activity were the same, we generated additional plots for each individual patient, which allowed us to visually compare the activity patterns recorded by each method. 

## 3. Results

Twenty-six participants or their family provided written consent. Data could not be collected from 5 participants; 4 were discharged and 1 had a deteriorating medical condition and was admitted to intensive care. Twenty-one participants completed measurement. The data of one participant could not be included in the analysis due to an error in the data file of the PAL2; therefore, analyses were based on the datasets of 20 participants. Demographic details of the 20 included participants are displayed in Table 1. 

**Figure 2:**
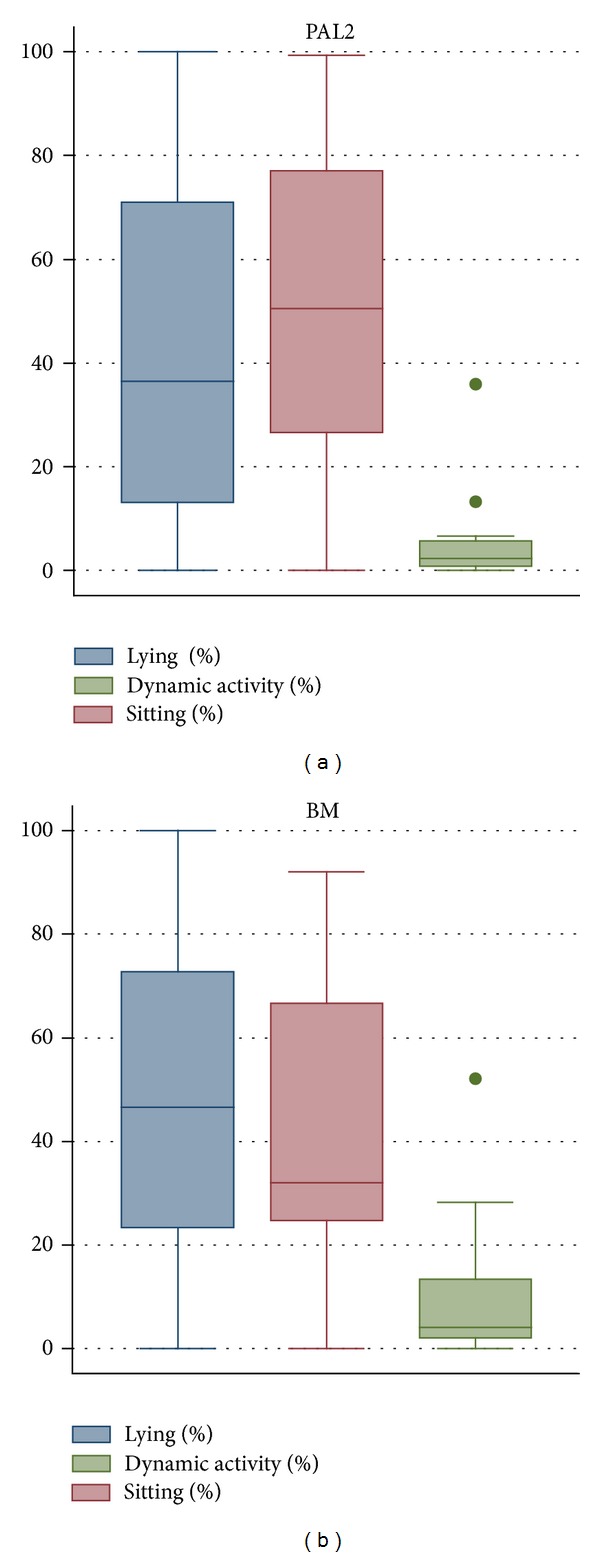
Median percentage of time spent in each category according to PAL2 and behavioural mapping. BM: behavioural mapping.

The median percentages of time per day spent in each activity category according to PAL2 and behavioural mapping are displayed in Figure 2. The median percentage and interquartile range (IQR) lying, sitting, and upright recorded by the PAL2 was 36% (15–68%), 51% (28–72%), and 2% (1–5%), respectively. The median percentages of lying, sitting, and upright recorded by behavioural mapping were 47% (24–72%), 32% (25–64%), and 4% (2–13%). 

### 3.1. Level of Agreement

Levels of agreement (ICC and CCC) and 95% confidence intervals are shown in Table 2. Correlations were very similar for the categories lying and upright for both ICC and CCC (ranging from 0.72 to 0.74). Correlation in the category sitting was somewhat lower, 0.68 for both ICC and CCC [24]. The strength of agreement was substantial for all categories. 

**Figure 3:**
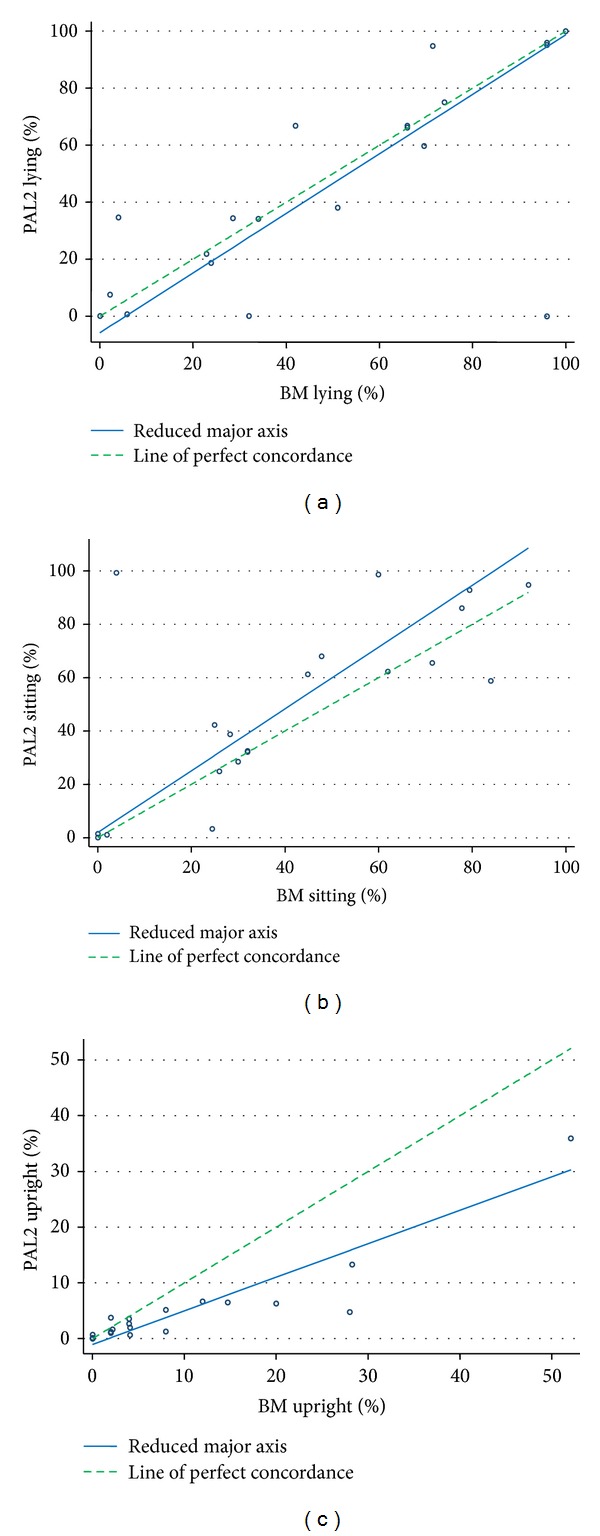
Scatterplots for each activity category including the line of perfect agreement and reduced major axis.

Scatterplots for each category, including the line of perfect agreement and reduced major axis, are shown in Figure 3. The very small intercepts showed that there was little evidence of fixed bias (i.e., there was no systematic difference in percentage of time spent between the two methods of measurement). In all three categories the data showed proportional bias. There is an indication of proportional bias when, for example, agreement gets smaller as the proportion of time spent in a given activity gets larger. Proportional bias was small for lying and sitting (slope = 1.04 and 1.15 resp.) and larger for upright (slope = 0.6). The more time participants spent upright, the bigger the difference became between the two methods, decreasing agreement. 

There was one notable outlier, where the data of the PAL2 showed that the participant spent 99% of the day sitting whereas behavioural mapping data showed that this participant spent 99% of the day lying and did not sit at all (see Figures 3(a) and 3(b)). Therefore, we conducted a post hoc analysis recalculating the level of agreement (ICC and CCC) with the data of the outlier removed. As expected the levels of agreement between PAL2 and behavioural mapping increased for the categories of lying and sitting (see Table 2).

The individual graphs of the PAL2 and behavioural mapping data showed similar patterns over the nine hour day for most participants. In one participant the data showed obvious discrepancy between the two methods; the participant was observed lying down, but the PAL2 recorded the participant as sitting. This was the same participant as the outlier identified in the scatterplots shown in Figure 3. Two other participants showed some discrepancy; in one the PAL2 recorded lying as sitting and in the other sitting was recorded as lying. Individual scatterplots of these participants are provided as Supplementary Materials available online at http://dx.doi.org/10.1155/2013/460482. 

### 3.2. Acceptability Wearing the PAL2

Of the 21 participants who wore the PAL2, only eight people were able to rate their experience on the Likert-scale. Thirteen people could not rate their experience, due to either cognitive impairments (*n* = 10), language (*n* = 2), or discharge before 5 p.m. (*n* = 1). Five participants strongly agreed, one participant was undecided and two disagreed with the statement “wearing the device on my leg was comfortable.” Three participants commented that the straps used to attach the device to the leg were too tight and therefore uncomfortable to wear. It was noted that one participant could not wear the device because of pain in the lower extremities. None of the participants who were monitored with the PAL2 took the device off during the monitoring day. No comments of nursing staff regarding the PAL2 were noted.

## 4. Discussion

Our results indicate that the PAL2 is an accurate tool to record activity early after stroke. We found substantial agreement between PAL2 data and data recorded by direct observation. Data from both methods showed that participants in acute stroke care spent a major part of the day inactive, lying in bed. Agreement in percentage of the day spent in different activities can give an indication of how well the PAL2 estimates activity overall, but we also wanted to know if the recordings of activity were time specific (i.e., at any given time, is the PAL2 recording the same activity as behavioural mapping?). We found that the PAL2 and observational mapping showed similar activity patterns over a day of observations in all but one patient.

Several accelerometers have been used and validated in a stroke population. A review of accelerometry based measures in stroke included 25 studies [15]. However none of the included studies looked at the ability of accelerometers to distinguish between lying, sitting, and upright and only three studies were conducted in an acute stoke population [18–20]. This study showed agreement between the two methods and visual inspection of the scatterplots showed similar patterns throughout the day. Some of the slight differences we found in recorded activities were expected, given the different sampling rates. When using behavioural mapping, it is assumed that the activity observed is continuous between consecutive observations. This assumption could have resulted in an overestimate of the observed activity compared to the recorded activity by the PAL2, especially of time spent upright. Upright activity in this study consisted of time spent standing and walking. In an acute setting one would expect that the majority of participants would not be able to stand or walk for long uninterrupted periods of time, certainly less than ten minutes. Therefore, a sampling rate of every ten minutes could have led to the higher estimate of time spent upright compared to the PAL2 data.

Overall the levels of activity of the participants were low and similar to the results of an observational study in an acute stroke population showing that participants in an Australian stroke unit spent over 50% of their active day (8 a.m.–5 p.m.) in bed [14]. Physical activity at a Norwegian stroke unit was higher, but substantial levels of bed rest remained [21]. In our study only 2% of their time was spent on activities that could help to improve recovery and mobility such as standing and walking. 

The datasets of the one outlier we identified showed that the PAL2 recorded the participant as sitting almost all day whereas behavioural mapping recorded that the participant was lying all day. This was also the case in one other participant but disagreement was less frequent. In one other patient the opposite occurred; the participant was observed as sitting whereas the PAL2 recorded the participant as lying down. As it was recorded by an observer, we can be confident that the behavioural mapping data is the true representation of the actual activity of that participant. The activity recorded by the PAL2 is determined by the settings of the threshold during the calibration process. When calibrating the PAL2 data the researcher needs to interpret the raw data. This is not always straightforward especially when participants are lying with their knees bent, as the PAL2 will record this as sitting, or in cases where participants are sitting with their legs straight, as the PAL2 will record this as lying down. 

We have discussed these issues with the producer of the device. In cases where a person has their legs straight in a horizontal plane the PAL2 will record the position as lying down. The recorded position is based on the combination of the information about the position of the upper and lower leg. No information is recorded about the position of the upper body, which is necessary to be able to distinguish sitting with straight legs from lying down. This may not be a major issue in acute stroke as it was rare for our participants to be sitting with their knees straight (seen in only 1 out of 20). Fortunately, in cases where the device records sitting when participants are actually lying in bed with their knees bent, a solution could be created to decrease measurement error of the device. A new version of the software was developed, which allows overwriting the recording of sitting in cases where you suspect the participant was lying with knees bent by setting an extra threshold. Hence instead of recording sitting, the device will record lying down in these particular instances. 

### 4.1. Study Limitations

We elected to record over a single day and this could be considered as a limitation of this study. Recording over a single day may not be representative of the “true” activity patterns of participants. Using a device in acute care can be more challenging than in other settings. The length of stay at an acute stroke ward is in most cases fairly short and monitoring over several days is often not feasible. The days that participants are admitted and discharged are not suitable to monitor. Furthermore, in the acute setting participants undergo multiple tests and some require the device to be taken off. That only leaves a few days during their hospital stay available for monitoring their physical activity. While behavioural mapping is a widely used method in a stroke setting, there is a need for an easier, less time consuming way to gather data on activity patterns in acute stroke care. Though we showed that both methods provide similar information on physical activity levels, behavioural mapping is not a perfect gold standard as a comparator method, since it has a low sampling rate. Confirmation of the accuracy of the PAL2 against video recording would be useful. The data are based on a small convenience sample, which limits external validity of the results. The inclusion criteria for this study were very broad, with no restrictions regarding cognitive function. This resulted in a large number of participants who could not communicate whether the device was comfortable and therefore the acceptability data should be interpreted with caution.

## 5. Conclusion

As other studies have shown, participants in acute stroke care are inactive. The PAL2 has an added advantage over other accelerometers by being able to distinguish between lying and sitting in an acute setting. We are not aware of any other devices that can reliably detect these changes in body position and that are tested in acute stroke.

## Supplementary Material

The Supplementary Material contains plots of observations and the PAL2 data of three participants. The plots illustrate examples of disagreement between the observations and PAL2 readings and include the data of the outlier.

## Figures and Tables

**Table 1:** Participant characteristics.

Variables	Sample *n* = 20 *n* (%)
Age (median IQR)	80 (76.5–83.5)
Gender	
Female	10 (50)
First ever stroke	16 (80)
Days since stroke (median, iqr)	7 (5–11)
Side of lesion	
Left	7 (35)
Right	13 (65)
Living arrangements	
Alone	8 (40)
With someone	12 (60)
Premorbid mobility	
Independent	14 (70)
With aid	5 (25)
Supervision	1 (5)
Stroke type: Oxfordshire	
TACI	5 (25)
PACI	7 (35)
POCI	3 (15)
LACI	2 (10)
Haemorrhage	3 (15)
Pre-morbid disability: Modified Rankin	
No symptoms	10 (50)
No significant disability	2 (10)
Slight disability	3 (15)
Moderate disability	4 (20)
Moderately severe disability	1 (5)
Severe disability	0 (0)
Stroke severity: NIHSS score	
Mild <8	12 (60)
Moderate 8–16	5 (25)
Severe >16	3 (15)

IQR: interquartile range. NIHSS: National Institutes of Health Stroke Scale, Total Anterior Circulation Infarct (TACI), Partial Anterior Circulation Infarct (PACI), Posterior Circulation Infarct (POCI), Lacunar Circulation Infarct (LACI).

**Table 2:** Intraclass and Concordance Correlation Coefficients (95% confidence intervals) between PAL2 and behavioural mapping.

Category	Complete set (*n* = 20)		Outlier excluded (*n* = 19)	
	ICC (95% CI)	CCC (95% CI)	ICC (95% CI)	CCC (95% CI)
Lying	0.74 (0.46–0.89)	0.73 (0.51–0.94)	0.92 (0.81–0.97)	0.92 (0.84–0.99)
Sitting	0.68 (0.36–0.86)	0.68 (0.44–0.91)	0.89 (0.75–0.96)	0.89 (0.79–0.98)
Upright	0.72 (0.43–0.88)	0.73 (0.58–0.87)	0.72 (0.41–0.88)	0.72 (0.57–0.87)
